# Supplementary material for: Anti-Leukemic Profiling of Oxazole-Linked Oxadiazole Derivatives: A Computational and Kinetic Approach
Source: Pharmaceuticals (Basel). 2025 Apr 25;18(5):625. doi: 10.3390/ph18050625 (PMC12114771; doi:10.3390/ph18050625)
Supplement: Supplementary file 1 [file pharmaceuticals-18-00625-s001.zip › pharmaceuticals-3600581-supplementary.pdf]

2.4 Cell Viability

In order to explore the living cancer cells after inhibition by synthesized potent compounds, cell viability analysis was conducted. This analysis shows that with increase in concentration of inhibitor the %age of active or living cancer cells got declined, this is due to the inhibitory effect of these inhibitors on cancer cells growth. At high concentration, inhibitor effects the cancer cells proliferation, reduced its progression rate which ultimately leads to the reduced number of cancer cells lines in affected areas. The nonlinear regression curve showing % cell viability for analog 6 was illustrated in Fig. S.1.

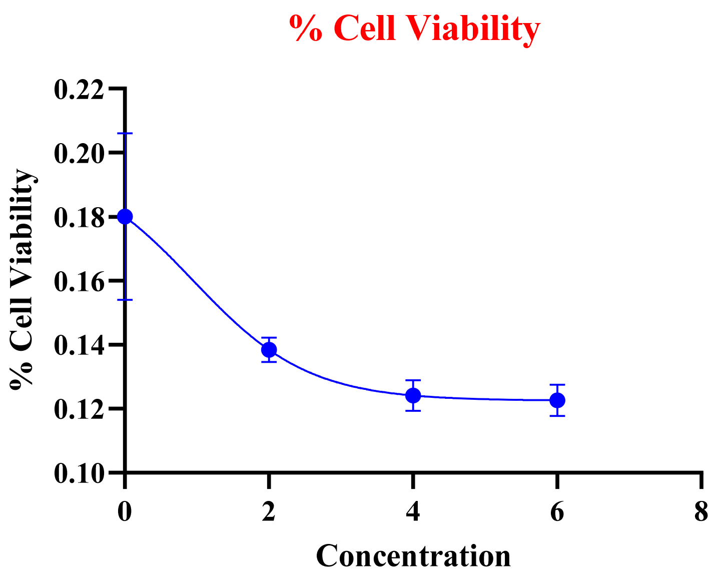

Figure S1. Represents the % cell viability for analog 6.

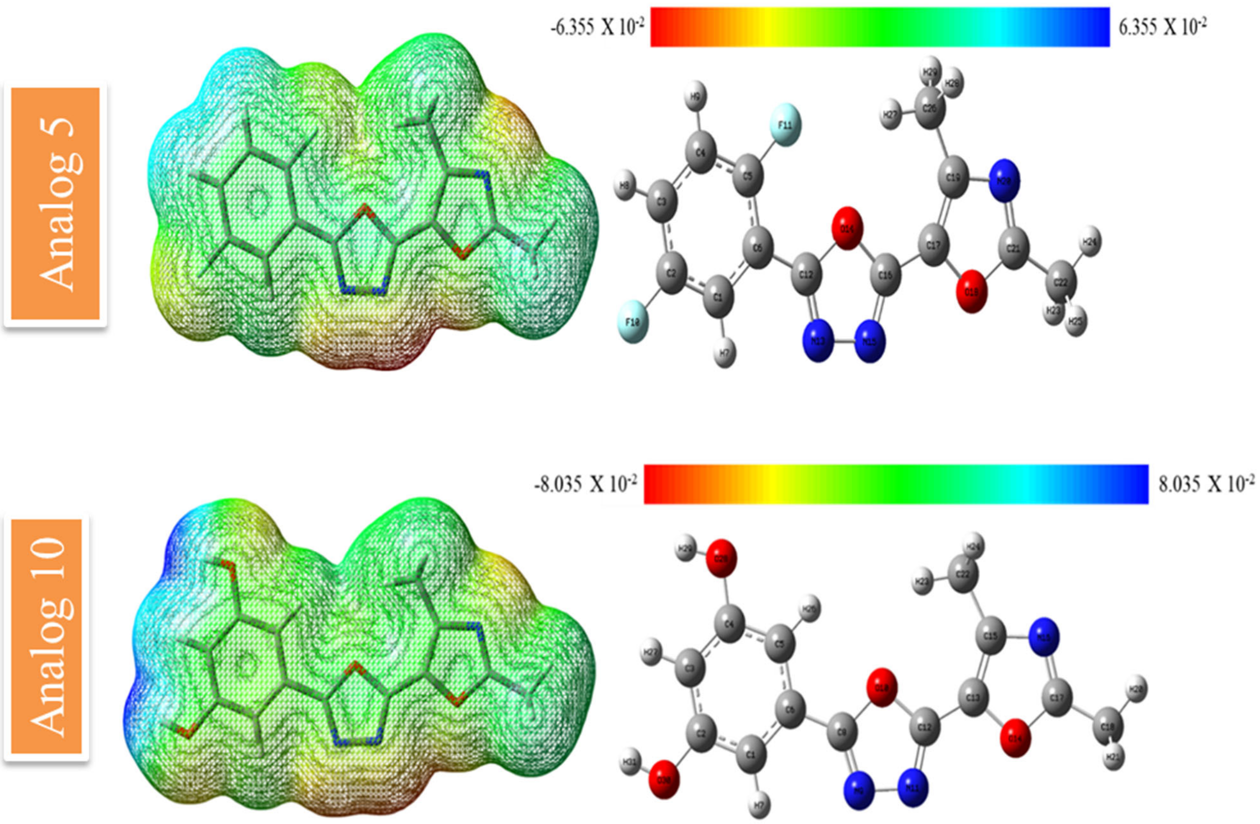

Figure S2. Electrostatic potential surfaces of analogs 5 and 10 showing the molecular arrangement and both electrophilic and nucleophilic region.

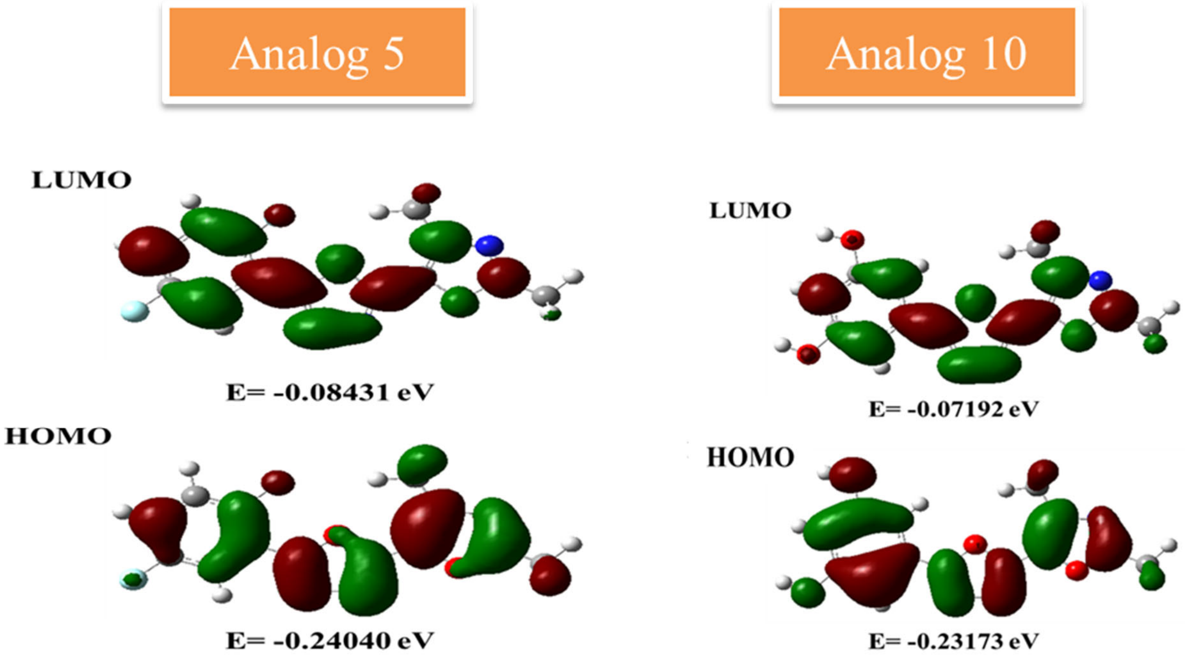

Figure S3. Molecular orbital analysis of analogs 5 and 10 visualizes orbital lobes, HOMO and LUMO orbitals, and their associated energy.

Graph-S1: This shows the drug-dose relationship against HL-60 by plotting the percentage inhibition of analog 5 versus the inhibitor concentration.

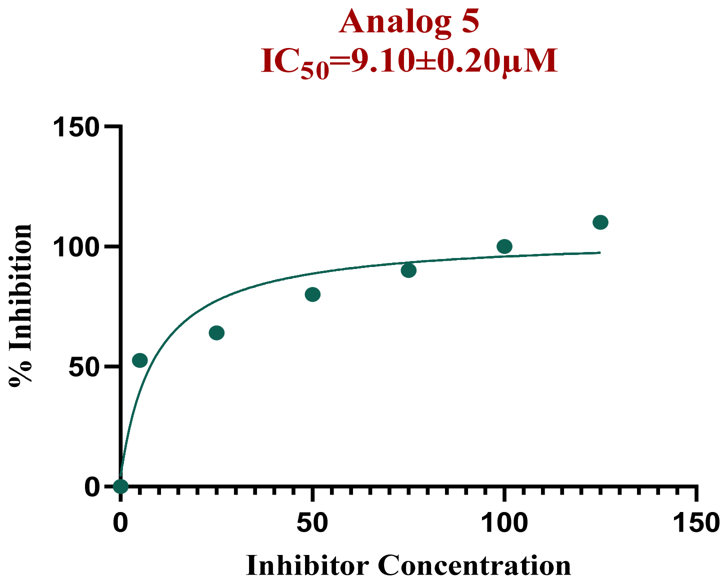

Graph-S2: This shows the drug-dose relationship against PLB-985 by plotting the percentage inhibition of analog 5 versus the inhibitor concentration.

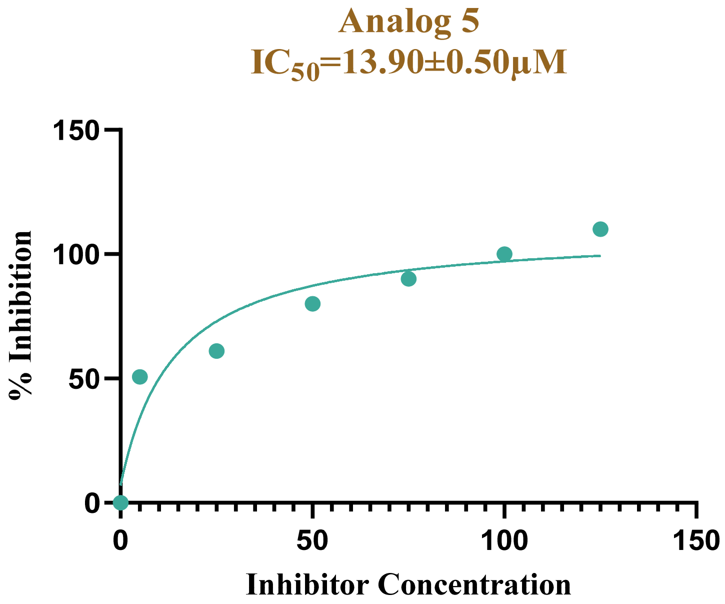

Graph-S3: This shows the drug-dose relationship against HL-60 by plotting the percentage inhibition of analog 10 versus the inhibitor concentration.

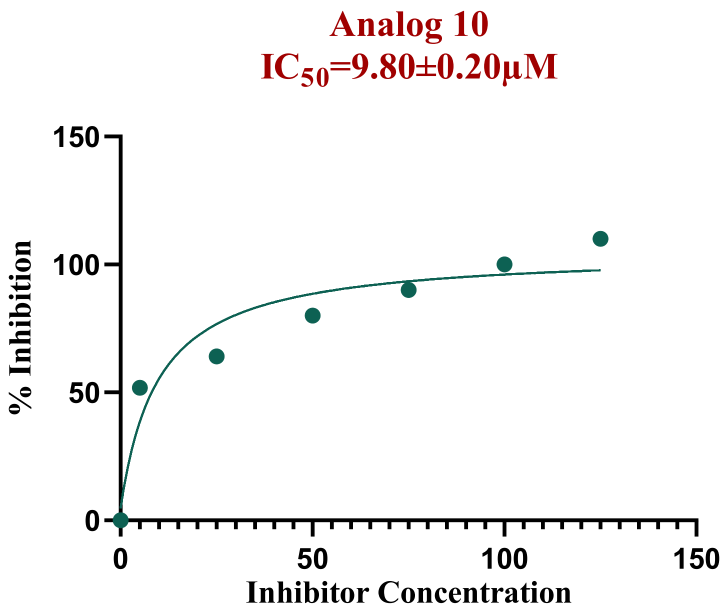

Graph-S4: This shows the drug-dose relationship against PLB-985 by plotting the percentage inhibition of analog 10 versus the inhibitor concentration.

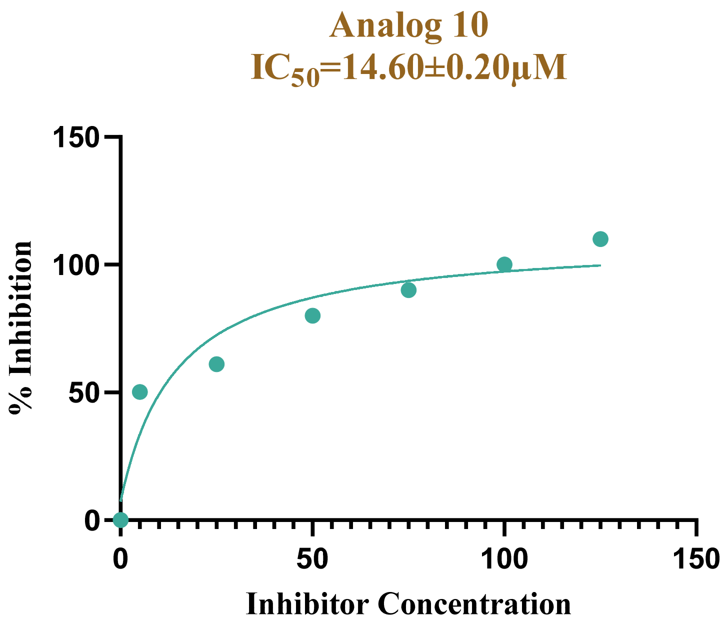

S1 Spectral Analysis

1. 2-(2,4-dimethyloxazol-5-yl)-5-(4-nitrophenyl)-1,3,4-oxadiazole

Yield: 78%, ^{1}H NMR (600MHz, DMSO-*d6*): *δ* 8.40 (d, *J* = 7.30 Hz, 2H, Ar-H), 8.20 (d, *J* = 7.27 Hz, 2H, Ar-H), 2.60 (s, 3H. H-aliphatic), 2.12 (s, 3H, H-aliphatic); ^{13}C NMR (150 MHz, DMSO-d6): *δ* 165.1, 164.2, 157.3, 147.6, 138.1, 132.1, 131.2, 130.7, 130.5, 128.7, 128.5, 14.3, 13.2; HREI MS:*m/z* calcd for $C_{13}H_{10}N_4O_4$ $[M]^+$ 286.25 Found 286.21.

2. 2-(4-chlorophenyl)-5-(2,4-dimethyloxazol-5-yl)-1,3,4-oxadiazole

Yield: 83%, ^{1}H NMR (600MHz, DMSO-*d6*): *δ* 7.70 (d, *J* = 7.25 Hz, 2H, Ar-H), 7.51 (d, *J* = 7.35 Hz, 2H, Ar-H), 2.59 (s, 3H, H-aliphatic), 2.11 (s, 3H, H-aliphatic); ^{13}C NMR (150 MHz, DMSO-d6): *δ* 165.2, 164.1, 157.2, 138.0, 134.2, 131.1, 129.3, 129.1, 128.7, 128.6, 124.1, 14.2, 13.3; HREI MS:*m/z* calcd for $C_{13}H_{10}ClN_3O_2$ $[M]^+$ 275.69 Found 275.65.

3. 2-(4-bromo-3-nitrophenyl)-5-(2,4-dimethyloxazol-5-yl)-1,3,4-oxadiazole

Yield: 72%, ^{1}H NMR (600MHz, DMSO-*d6*): *δ* 8.43 (s, 1H, Ar-H), 8.09 (d, *J* = 8.88 Hz, 1H, Ar-H), 7.95 (d, *J* = 7.56 Hz, 1H, Ar-H), 2.60 (s, 3H, H-aliphatic), 2.13 (s, 3H, H-aliphatic); ^{13}C NMR (150 MHz, DMSO-d6): *δ* 165.1, 164.3, 157.4, 150.7, 138.5, 134.5, 133.8, 131.6, 126.6, 124.3, 118.7, 14.6, 13.6; HREI MS:*m/z* calcd for $C_{13}H_9BrN_4O_4$ $[M]^+$ 365.14 Found 365.10.

4. 2-(4-bromo-2-nitrophenyl)-5-(2,4-dimethyloxazol-5-yl)-1,3,4-oxadiazole

Yield: 69%, ^{1}H NMR (600MHz, DMSO-*d6*): *δ* 8.62 (s, 1H, Ar-H), 8.07 (d, *J* = 6.66 Hz, 1H, Ar-H), 7.74 (d, *J* = 8.10 Hz, 1H, Ar-H), 2.77 (s, 3H, H-aliphatic), 2.16 (s, 3H, H-aliphatic); ^{13}C NMR (150 MHz, DMSO-d6): *δ* 165.8, 164.7, 157.8, 147.6, 138.8, 138.5, 131.6, 130.8, 130.5, 126.6, 122.3, 14.6, 13.2; HREI MS:*m/z* calcd for $C_{13}H_9BrN_4O_4$ $[M]^+$ 365.14 Found 365.10.

5. 2-(2,5-difluorophenyl)-5-(2,4-dimethyloxazol-5-yl)-1,3,4-oxadiazole

Yield: 79%, ^{1}H NMR (600MHz, DMSO-*d6*): *δ* 7.48 (s, 1H, Ar-H), 7.33 (d, *J* = 7.26 Hz, 1H, Ar-H), 7.22 (d, *J* = 7.86 Hz, 1H, Ar-H), 2.71 (s, 3H, H-aliphatic), 2.14 (s, 3H, H-aliphatic); ^{13}C NMR (150 MHz, DMSO-d6): *δ* 165.5, 164.2, 157.8, 157.6, 153.7, 138.5, 131.6, 125.5, 118.7, 118.6, 114.3, 14.6, 13.2; HREI MS:*m/z* calcd for $C_{13}H_9F_2N_3O_2$ $[M]^+$ 277.23 Found 277.19.

6. 2-(2,4-dimethyloxazol-5-yl)-5-(4-(trifluoromethyl)phenyl)-1,3,4-oxadiazole

Yield: 78%, ^{1}H NMR (600MHz, DMSO-d_6): δ 7.92 (d, J = 7.37 Hz, 2H, Ar-H), 7.64 (d, J = 7.30 Hz, 2H, Ar-H), 2.63 (s, 3H, H-aliphatic), 2.15 (s, 3H, H-aliphatic); ^{13}C NMR (150 MHz, DMSO-d$_6$): δ 165.4, 164.2, 157.8, 148.8, 138.8, 131.6, 131.5, 127.6, 127.3, 125.5, 125.1, 124.7, 14.6, 13.2; HREI MS:*m/z* calcd for $C_{14}H_{10}F_3N_3O_2$ $[M]^+$ 309.25 Found 309.21.

7. 4-(5-(2,4-dimethyloxazol-5-yl)-1,3,4-oxadiazol-2-yl)-3-fluorophenol

Yield: 71%, ^{1}H NMR (600MHz, DMSO-d_6): δ 9.44 (s, 1H, H-OH), 7.58 (d, J = 8.16 Hz, 1H, Ar-H), 7.38 (s, 1H, Ar-H), 7.13 (d, J = 7.86 Hz, 1H, Ar-H), 2.71 (s, 3H, H-aliphatic), 2.14 (s, 3H, H-aliphatic); ^{13}C NMR (150 MHz, DMSO-d$_6$): δ 165.8, 164.4, 160.4, 158.3, 157.8, 138.2, 131.6, 130.7, 116.3, 112.8, 104.5, 14.3, 13.2; HREI MS:*m/z* calcd for $C_{13}H_{10}FN_3O_3$ $[M]^+$ 275.24 Found 275.20.

8. 2-(3-chloro-4-fluorophenyl)-5-(2,4-dimethyloxazol-5-yl)-1,3,4-oxadiazole

Yield: 75%, ^{1}H NMR (600MHz, DMSO-d_6): δ 8.13 (d, J = 7.14 Hz, 1H, Ar-H), 7.83 (s, 1H, Ar-H), 7.38 (d, J = 8.16 Hz, 1H, Ar-H), 2.78 (s, 3H, H-aliphatic), 2.13 (s, 3H, H-aliphatic); ^{13}C NMR (150 MHz, DMSO-d$_6$): δ 165.7, 164.7, 158.3, 157.8, 138.2, 131.6, 128.7, 125.5, 122.3, 121.1, 117.1, 14.3, 13.6; HREI MS:*m/z* calcd for $C_{13}H_9ClFN_3O_2$ $[M]^+$ 293.68 Found 293.64.

9. 2-(2,4-dimethyloxazol-5-yl)-5-(4-fluorophenyl)-1,3,4-oxadiazole

Yield: 68%, ^{1}H NMR (600MHz, DMSO-d_6): δ 8.32 (d, J = 7.44 Hz, 2H, Ar-H), 7.43 (d, J = 7.44 Hz, 2H, Ar-H), 2.73 (s, 3H, H-aliphatic), 2.18 (s, 3H, H-aliphatic); ^{13}C NMR (150 MHz, DMSO-d$_6$): δ 165.5, 164.2, 162.2, 157.8, 138.2, 131.6, 128.5, 128.3, 121.1, 116.4, 116.1, 14.3, 13.6; HREI MS:*m/z* calcd for $C_{13}H_{10}FN_3O_2$ $[M]^+$ 259.24 Found 259.20.

10. 5-(5-(2,4-dimethyloxazol-5-yl)-1,3,4-oxadiazol-2-yl)benzene-1,3-diol

Yield: 73%, ^{1}H NMR (600MHz, DMSO-d_6): δ 9.43 (s, 2H, H-OH), 6.80 (s, 2H, Ar-H), 7.35 (d, J = 7.36 Hz, 1H, Ar-H), 2.61 (s, 3H, H-aliphatic), 2.13 (s, 3H, H-aliphatic); ^{13}C NMR (150 MHz, DMSO-d$_6$): δ 165.2, 164.3, 158.7, 158.5, 157.2, 138.2, 131.3, 128.6, 105.4, 105.2, 103.2, 14.3, 13.4; HREI MS:*m/z* calcd for $C_{13}H_{11}N_3O_4$ $[M]^+$ 273.25 Found 273.21.

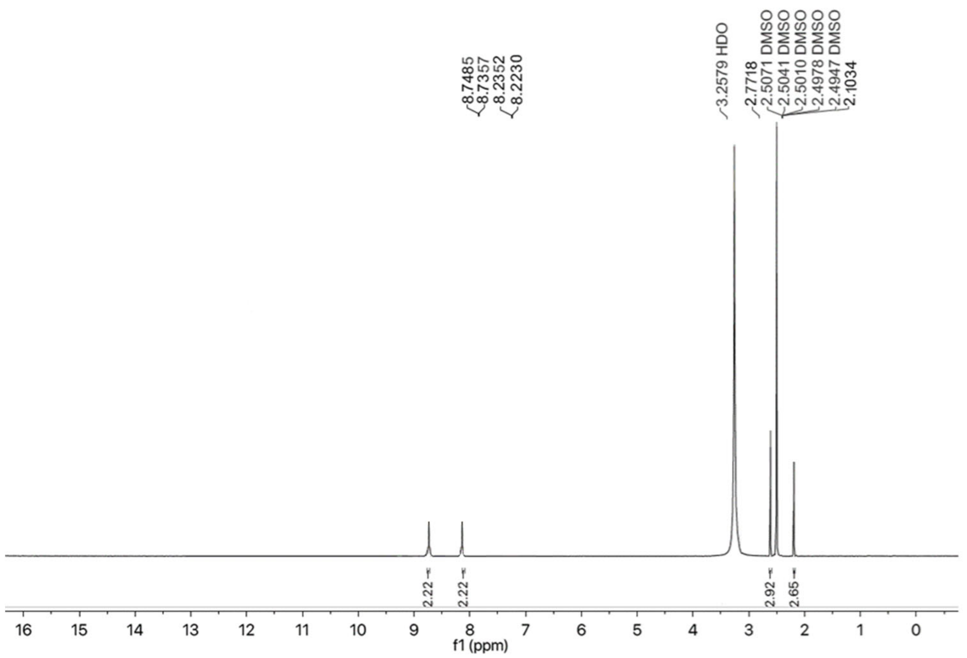

Figure S4. ^{1}H-NMR analysis of compound-1.

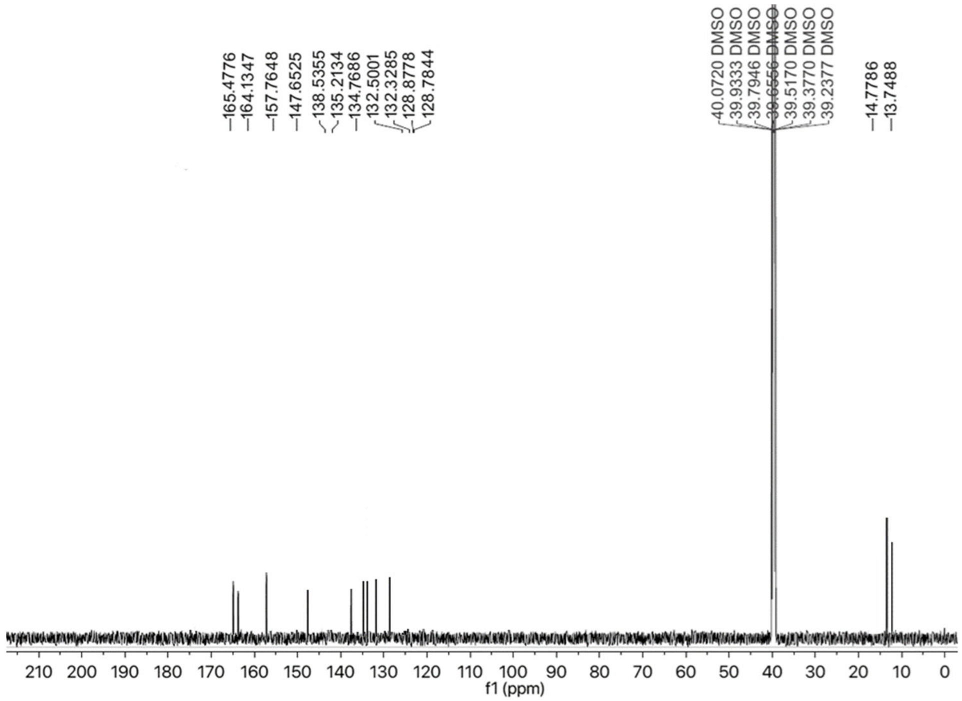

Figure S5. ^{13}C-NMR analysis of compound-1.

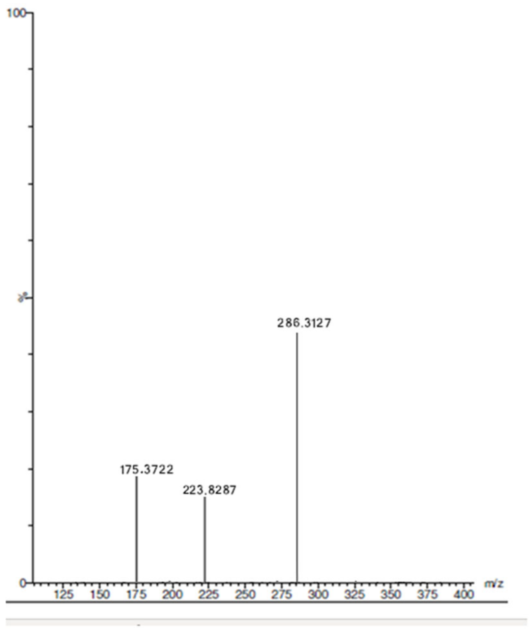

Figure S6. HREI-MS analysis of compound-1.

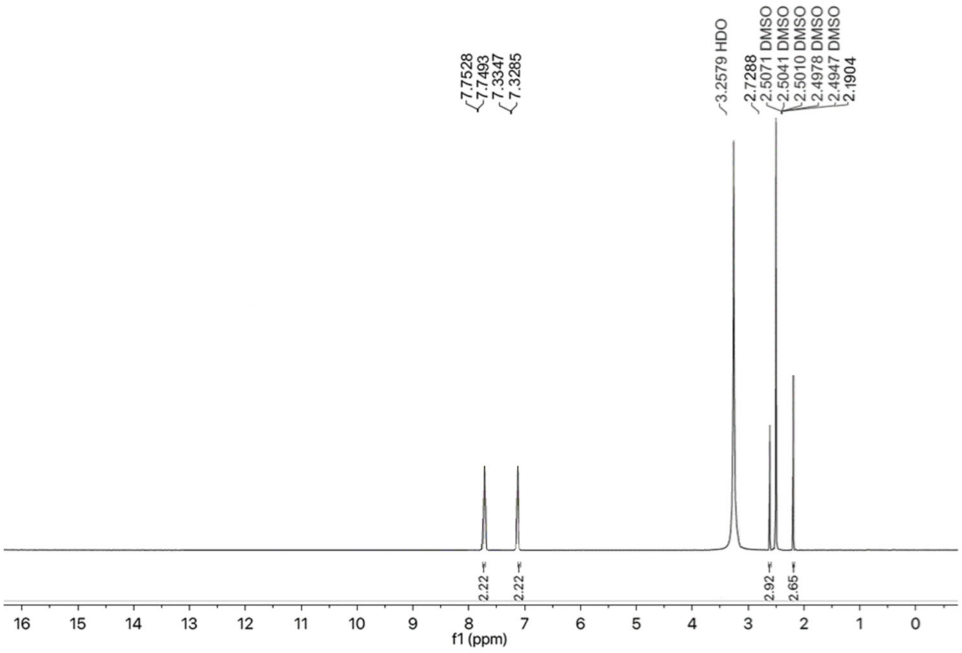

Figure S7. ^{1}H-NMR analysis of compound-2.

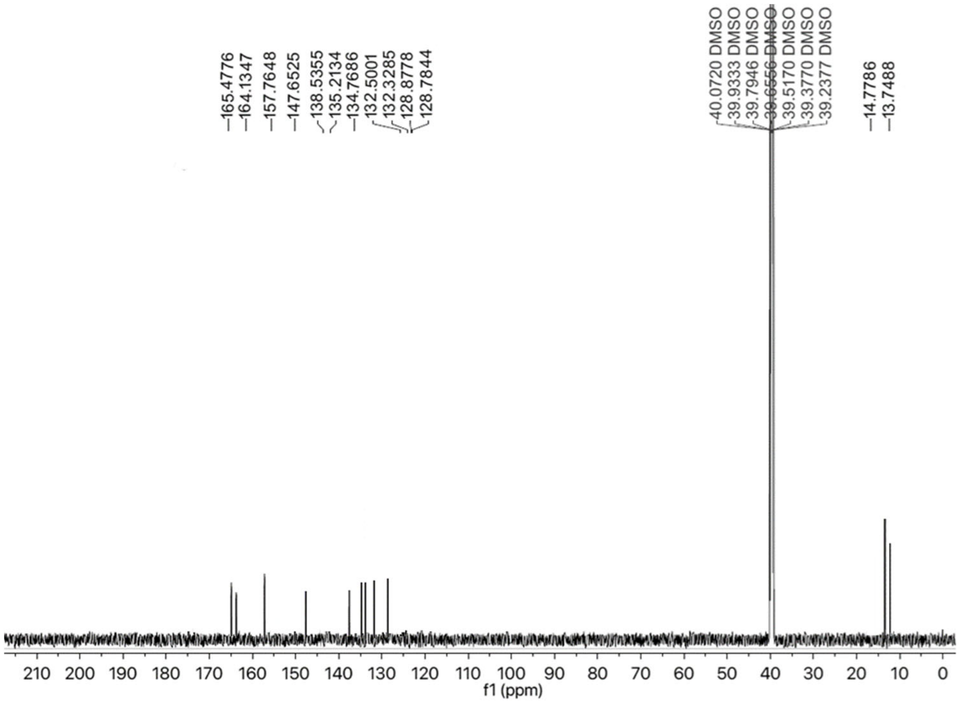

Figure S8. ^{13}C-NMR analysis of compound-2.

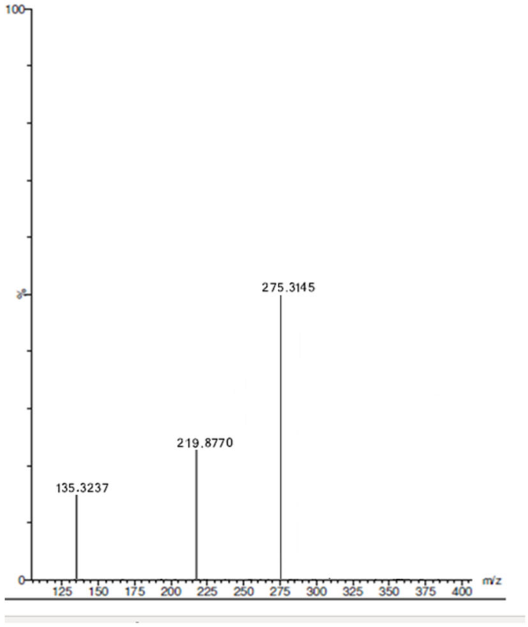

Figure S9. HREI-MS analysis of compound-2.

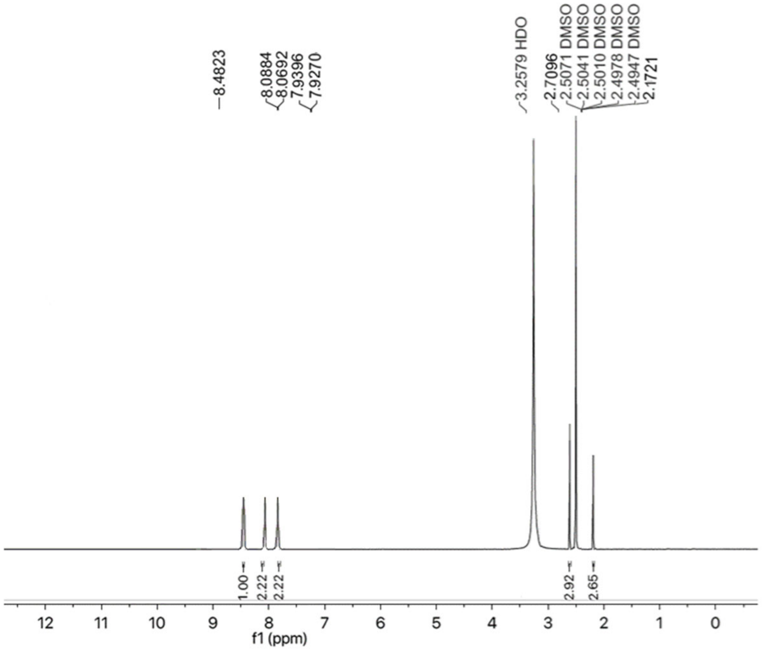

Figure S10. ^{1}H-NMR analysis of compound-3.

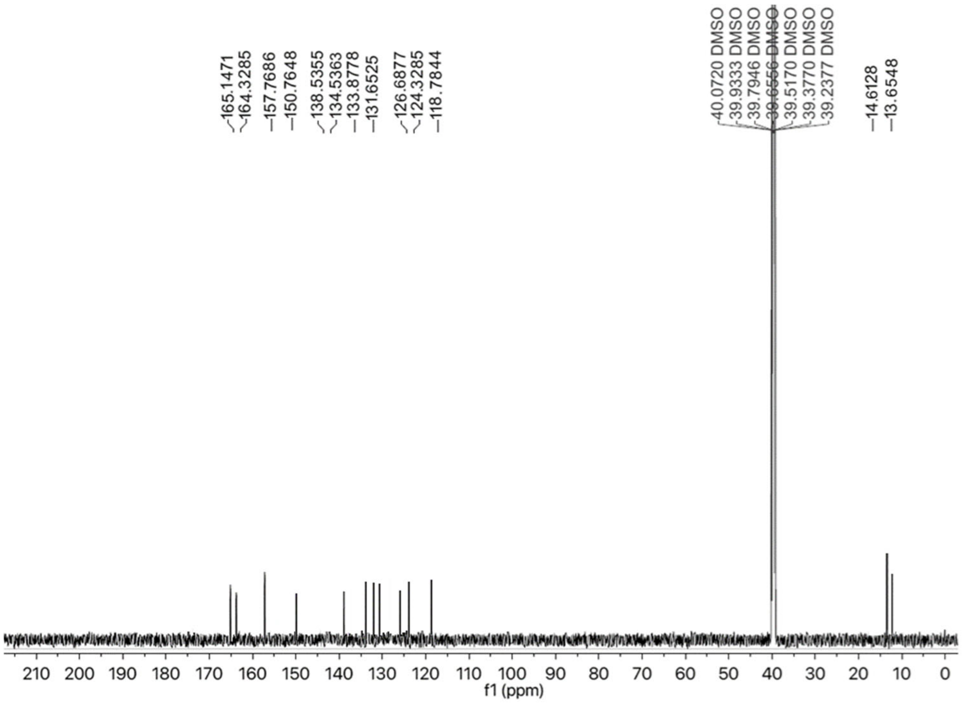

Figure S11. ^{13}C-NMR analysis of compound-3.

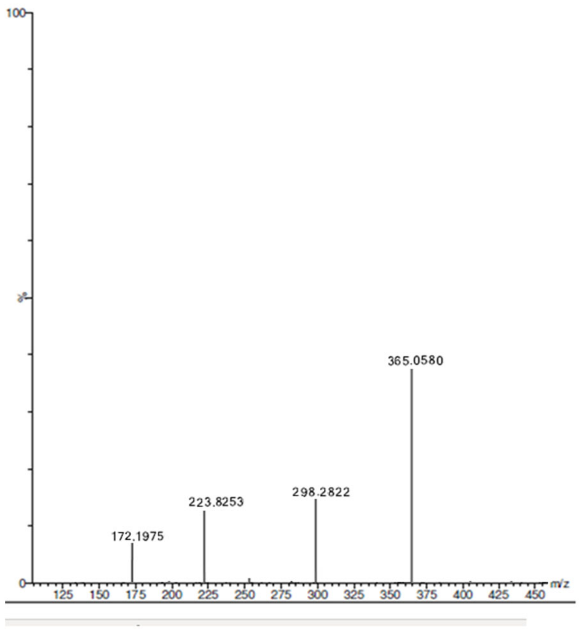

Figure S12. HREI-MS analysis of compound-3.

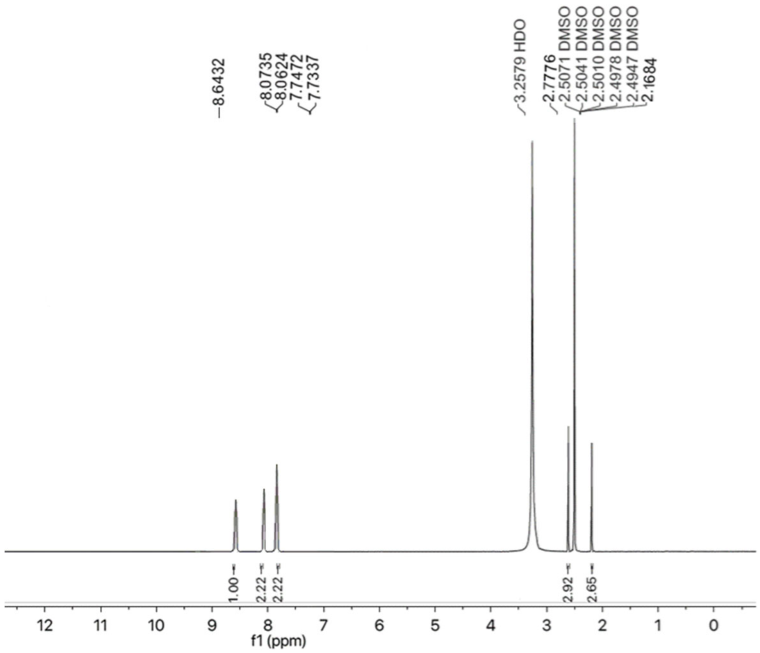

Figure S13. ^{1}H-NMR analysis of compound-4.

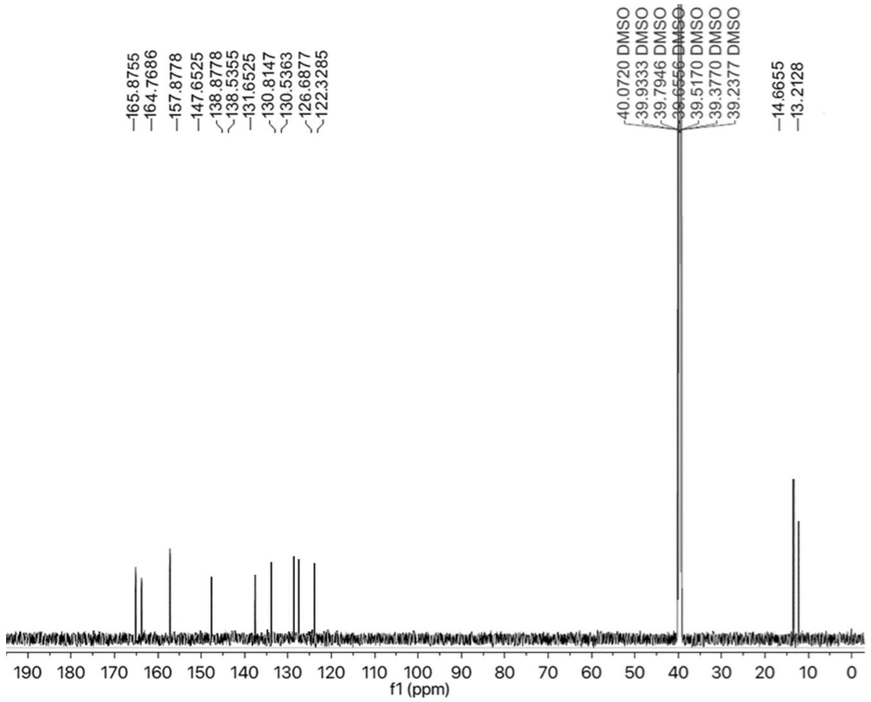

Figure S14. ^{13}C-NMR analysis of compound-4.

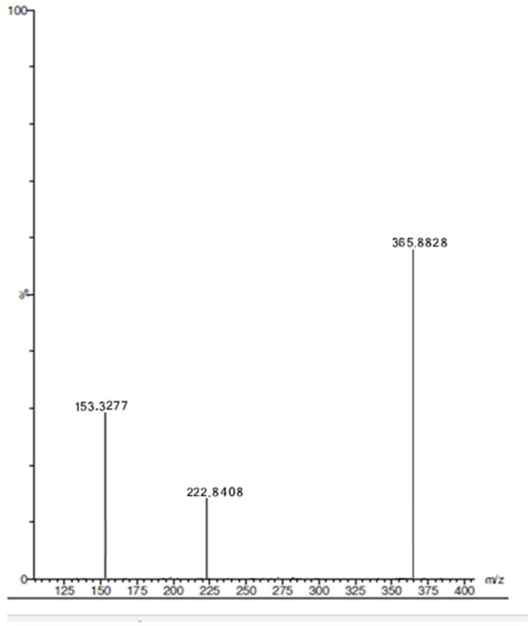

Figure S15. HREI-MS analysis of compound-4.

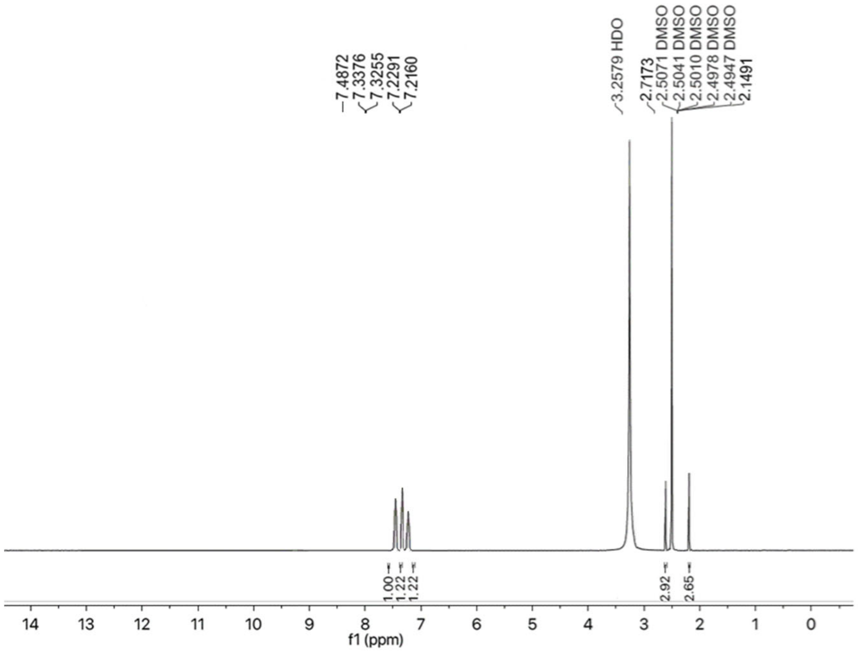

Figure S16. ^{1}H-NMR analysis of compound-5.

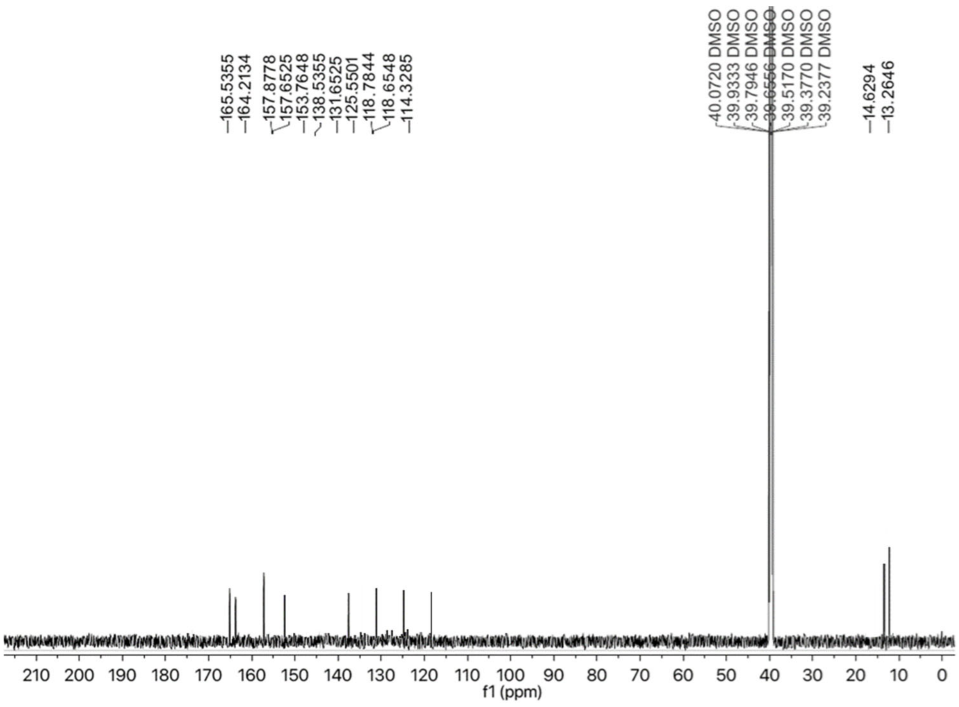

Figure S17. ^{13}C-NMR analysis of compound-5.

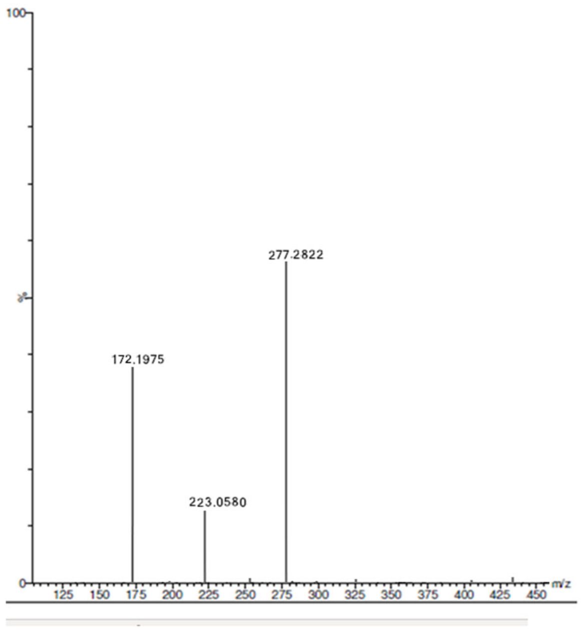

Figure S18. HREI-MS analysis of compound-5.

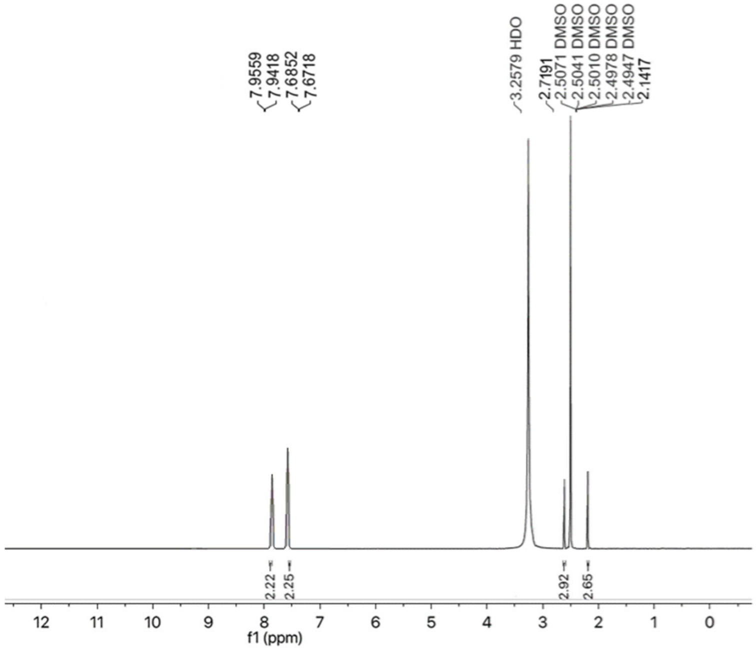

Figure S19. ^{1}H-NMR analysis of compound-6.

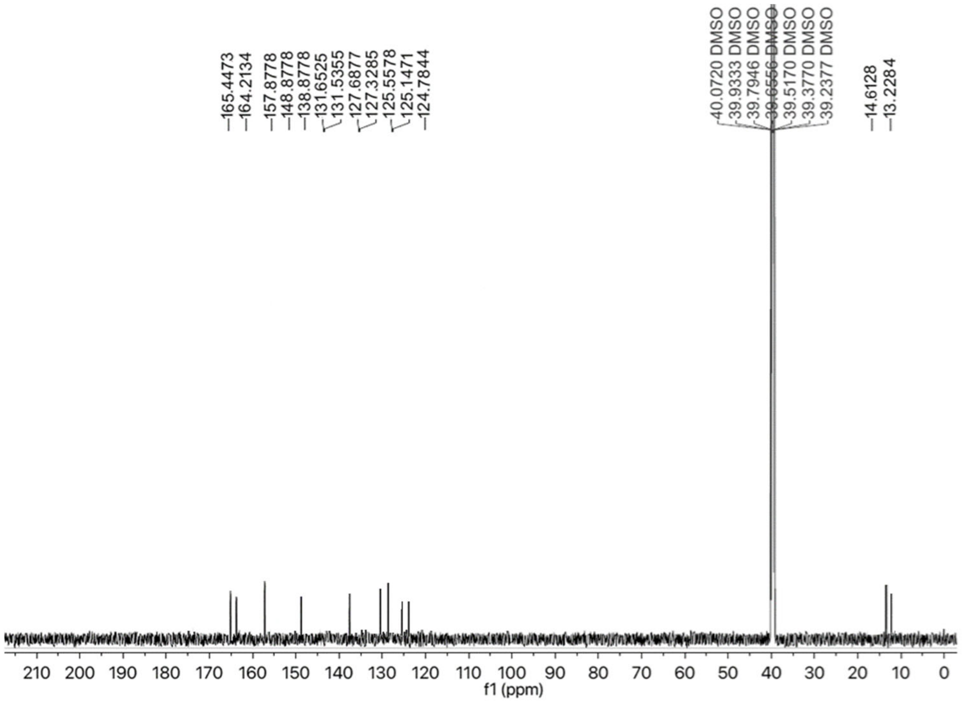

Figure S20. ^{13}C-NMR analysis of compound-6.

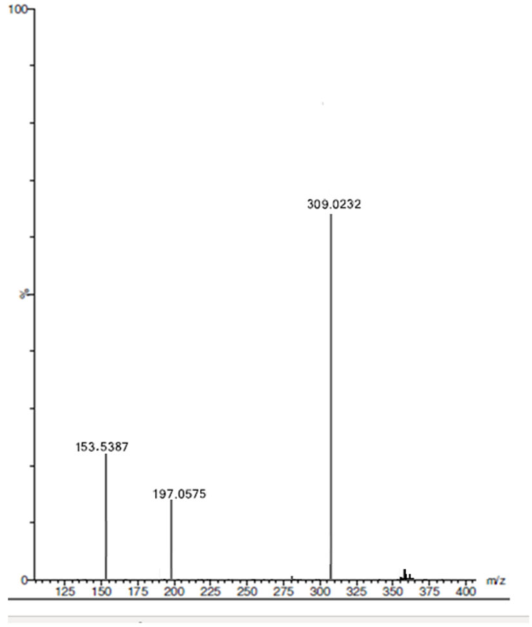

Figure S21. HREI-MS analysis of compound-6.

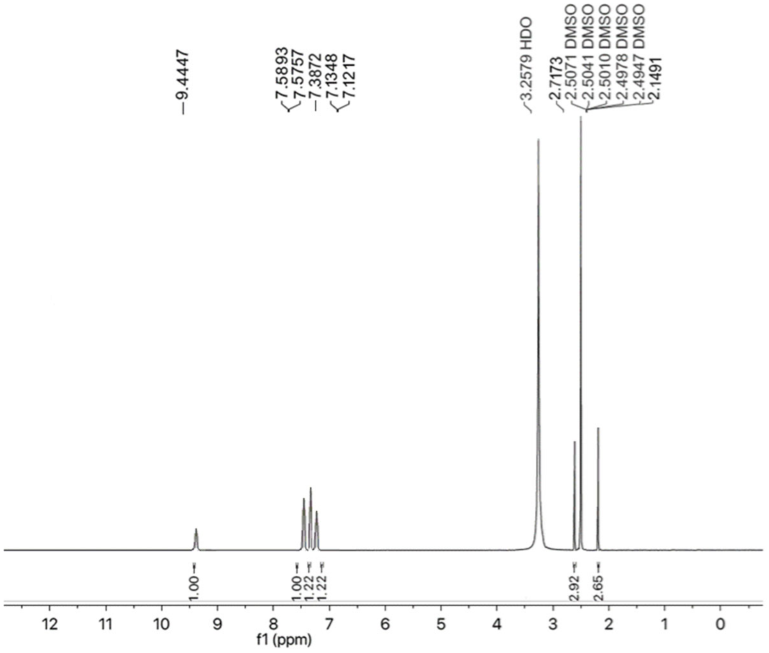

Figure S22. ^{1}H-NMR analysis of compound-7.

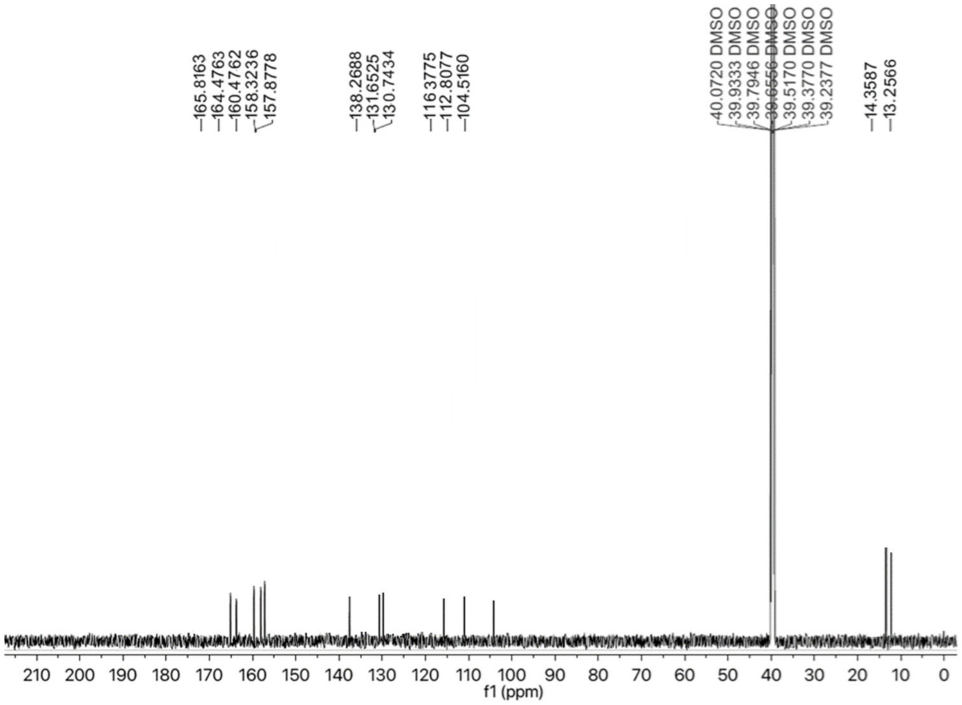

Figure S23. ^{13}C-NMR analysis of compound-7.

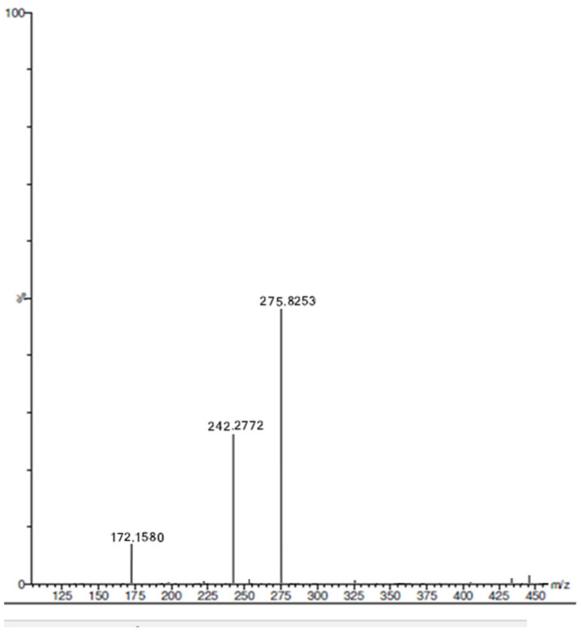

Figure S24. HREI-MS analysis of compound-7.

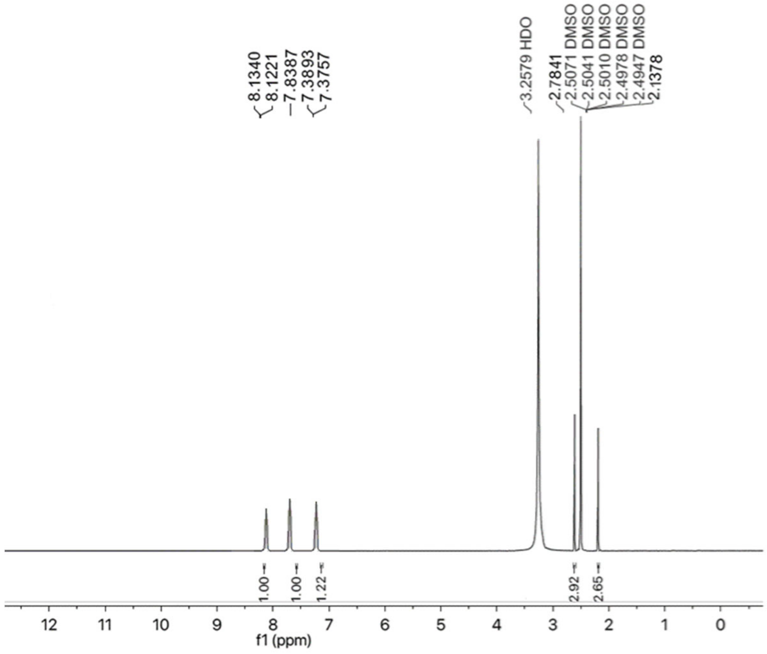

Figure S25. ^{1}H-NMR analysis of compound-8.

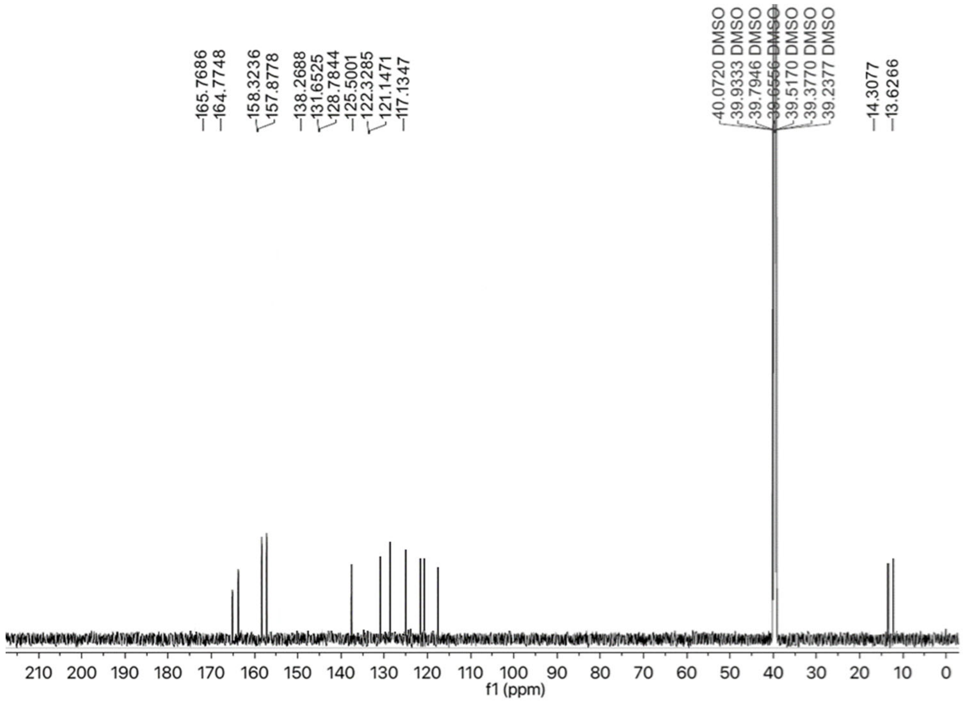

Figure S26. ^{13}C-NMR analysis of compound-8.

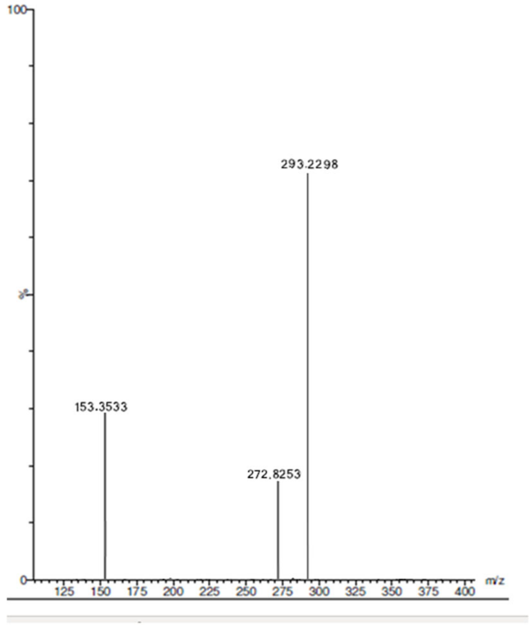

Figure S27. HREI-MS analysis of compound-8.

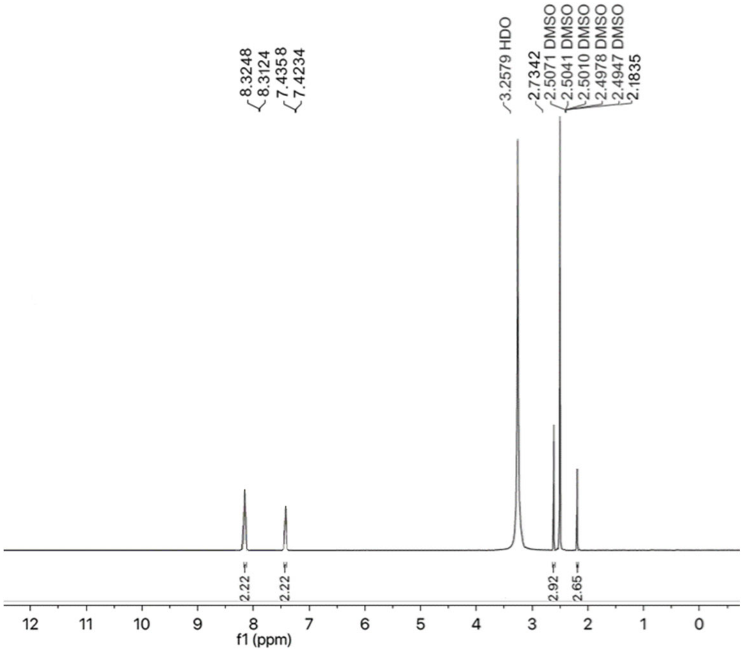

Figure S28. ^{1}H-NMR analysis of compound-9.

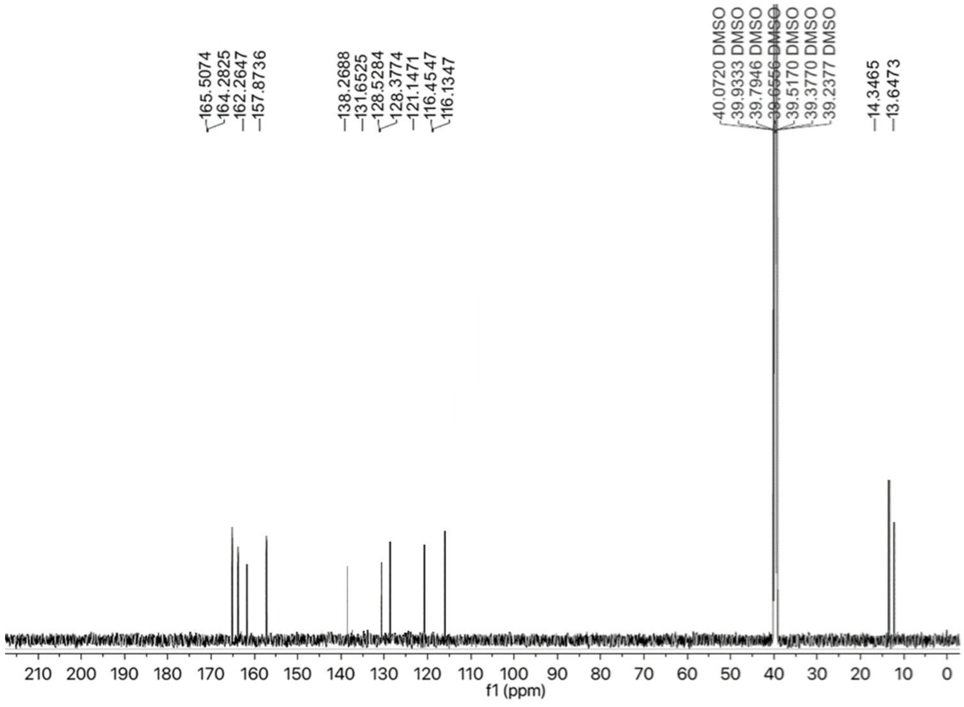

Figure S29. ^{13}C-NMR analysis of compound-9.

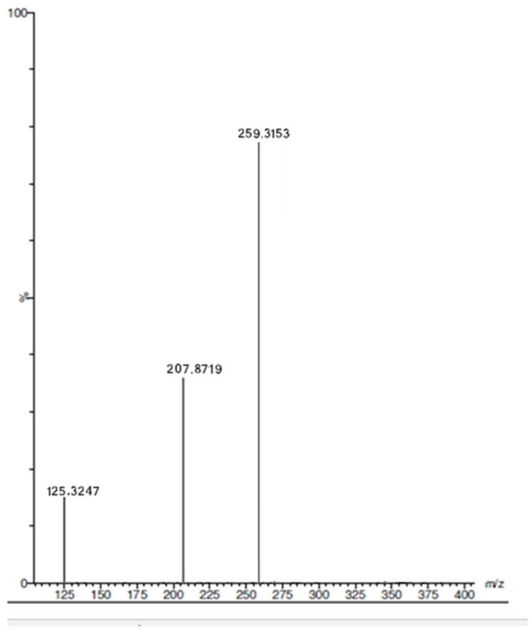

Figure S30. HREI-MS analysis of compound-9.

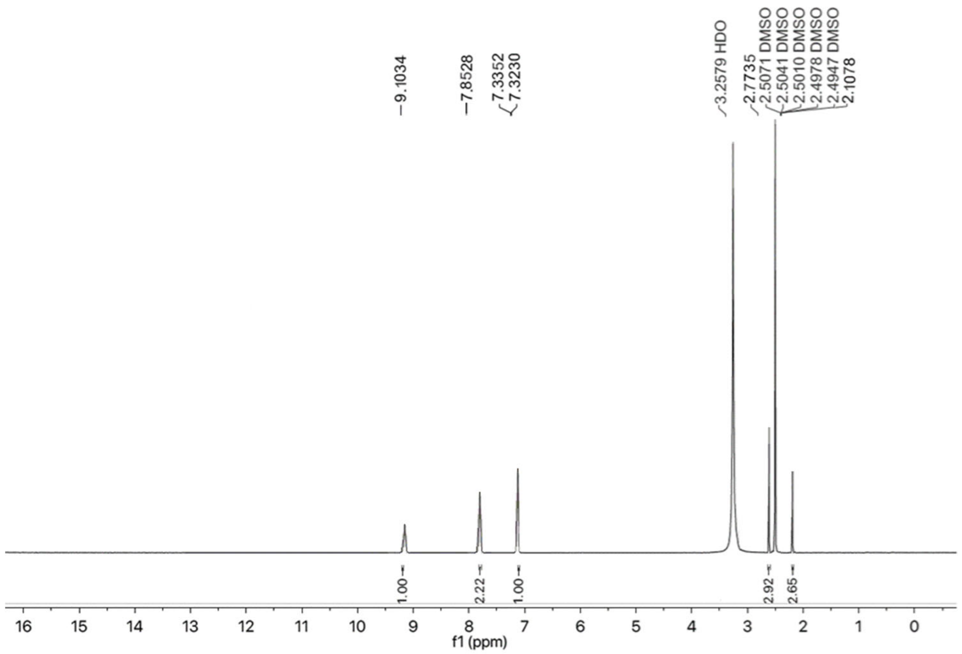

Figure S31. ^{1}H-NMR analysis of compound-10.

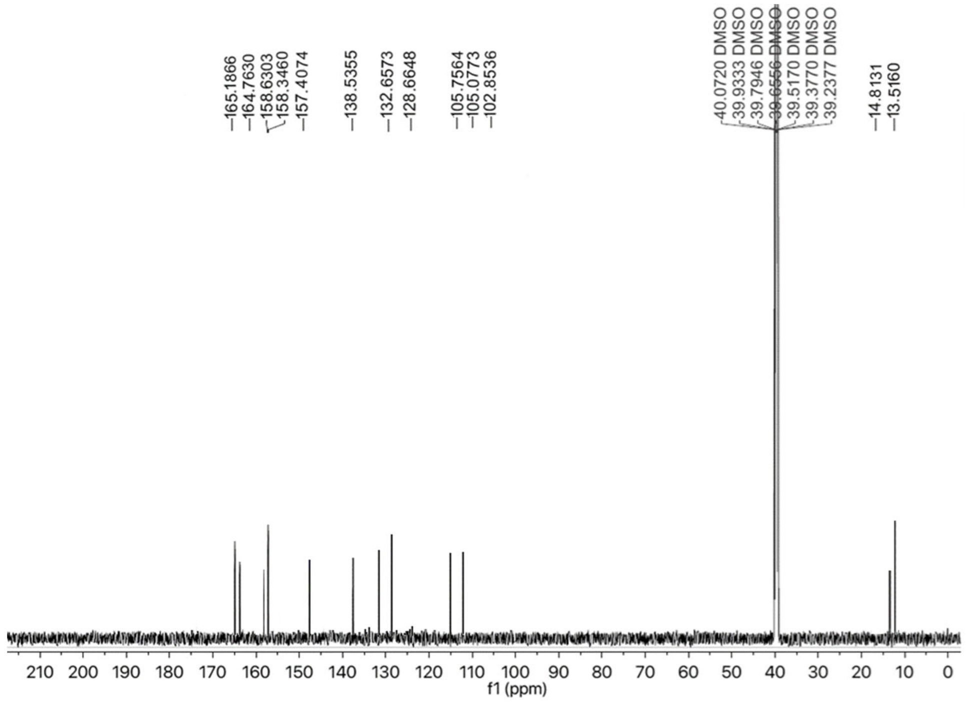

Figure S32. ^{13}C-NMR analysis of compound-10.

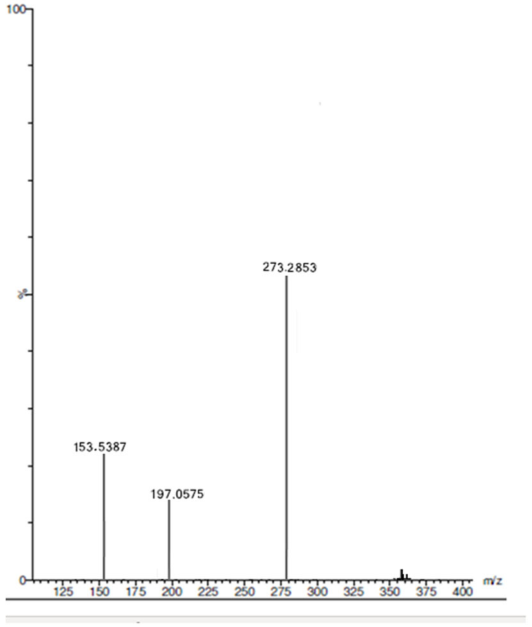

Figure S33. HREI-MS analysis of compound-10.

S.2 Molecular docking protocol

For the docking studies, the crystal structure of human BACE1 was retrieved from Protein Data Bank (PDB ID: 2QP8) and prepared using MOE [39]. The chain B and water molecules were removed from the system and the receptor structure was protonated (at pH 6.0 and 300 K) using the Protonate 3D tool and hydrogen atoms were also added. OPLS-AA force field [37] was used to assign atom types and partial charges in the receptor structure, which was further energy-minimized using the same force field. The active residues were defined by considering the binding site residues, previously identified by the Site Finder tool on MOE package. All compounds were docked in the given binding site. Next, the protein–ligand interaction fingerprinting (PLIF) module in MOE was applied to fingerprint the binding interactions of crucial residues between the compounds and the protein. Additional associations and poses of binding were visually studied using MOE [40].

S.3 DFT assay protocol

The geometric parameters and energies were computed by density functional theory at the B3LYP/CEP-31G level of theory, using the GAUSSIAN 98W package of the programs [41], on geometries that were optimized at CEP-31G basis set. The high basis set was chosen to detect the energies at a highly accurate level. The atomic charges were computed using the natural atomic orbital populations. The B3LYP is the key word for the hybrid functional [42], which is a linear combination of the gradient functionals proposed by Becke [43] and Lee, Yang and Parr [44], together with the Hartree-Fock local exchange function [45]. UV spectra were recorded in Rigol, Ultra- 3000 series in Enzymology and Fungal Biotechnology Lab, Faculty of Science, Zagazig University.

S.4 Enzyme kinetic studies

The kinetic studies were performed by using varying concentrations (µM) which were pre-incubated with test enzymes (250 µl) (3 U/ml) solution at 37°C for 10 minutes. In another set of test tubes, test enzyme was pre-incubated with phosphate buffer (250 µl) (100 mM, pH 6.8), then varying concentrations (0.0-8.0 mM) starch was added to both reaction mixtures which were incubated at 37°C for 10 minutes. Afterward, DNS (100 µl) was added to the mixtures for quenching the reaction and the mixtures boiled for 10 minutes then absorbance was measured

using a spectrometer at 540 nm using a maltose standard curve and converted to reaction velocities. [46].

39. Molecular Operating Environment (MOE), version 2016.08; Chemical Computing Group ULC: Montreal, QC, Canada, (2016).
40. W.L. Jorgensen, D.S. Maxwell, J. Tirado-Rives, Development and Testing of the OPLS All-Atom Force Field on Conformational Energetics and Properties of Organic Liquids, J. Am. Chem. Soc. 118 (1996) 11225–11236.
41. Molecular Operating Environment (MOE). 2015.10; Chemical Computing Group ULC, 1010 Sherbooke St. West, Suite #910 Montreal, QC, Canada, H3A 2R7. (2015).
42. M.J. Frisch, Gaussian 98, Revision A.9; Gaussian INc.: Pittsburgh, PA, USA. 1998.
43. A.F. El-Baz, N.M. Sorour, Y.M. Shetaia, Trichosporon jirovecii-mediated synthesis of cadmium sulfide nanoparticles, J. Basic Microbiol. 56 (2016) 520–530.
44. A.D. Becke, Density-functional exchange-energy approximation with correct asymptotic behavior, Phys. Rev. 38 (1988) 3098–3100.
45. C. Lee, W. Yang, R.G. Parr, Development of the Colle-Salvetti correlation-energy formula into a functional of the electron density, Phys. Rev. B. 37 (1988) 785–789.
46. R.L. Flurry, Jr. Molecular Orbital Theory of Bonding in Organic Molecules; Marcel Dekker: New York, NY, USA. (1968).
47. Singh P, Seboletswe P, Kumar G, Gcabashe N, Olofinsan K, Idris A, Islam S. Benzylidenehydrazine Derivatives: Synthesis, Antidiabetic Evaluation, Antioxidation, Mode Of Inhibition, DFT And Molecular Docking Studies. Chemistry & Biodiversity.:e202401556.
